# Nature of Acquired Immune Responses, Epitope Specificity and Resultant Protection from SARS-CoV-2

**DOI:** 10.3390/jpm11121253

**Published:** 2021-11-25

**Authors:** Reginald M. Gorczynski, Robyn A. Lindley, Edward J. Steele, Nalin Chandra Wickramasinghe

**Affiliations:** 1 Institute of Medical Science, Department of Immunology and Surgery, University of Toronto, Toronto, ON M5S 3G3, Canada; 2 Department of Clinical Pathology, Faculty of Medicine, Dentistry & Health Sciences, University of Melbourne, Melbourne, VIC 3000, Australia; robyn.lindley@unimelb.edu.au; 3 GMDx Group Ltd., Melbourne, VIC 3000, Australia; 4 C.Y.O’Connor ERADE Village Foundation, Piara Waters, Perth, WA 6207, Australia; e.j.steele@bigpond.com; 5 Melville Analytics Pty Ltd., Melbourne, VIC 3000, Australia; 6 Buckingham Centre for Astrobiology, University of Buckingham, Buckingham MK18 1EG, UK; NCWick@gmail.com; 7 Centre for Astrobiology, University of Ruhuna, Matara 81000, Sri Lanka; 8 National Institute of Fundamental Studies, Kandy 20000, Sri Lanka

**Keywords:** SARS-CoV-2, host resistance, innate immunity, acquired immunity, mucosal immunity, vaccination

## Abstract

The primary global response to the SARS-CoV-2 pandemic has been to bring to the clinic as rapidly as possible a number of vaccines that are predicted to enhance immunity to this viral infection. While the rapidity with which these vaccines have been developed and tested (at least for short-term efficacy and safety) is commendable, it should be acknowledged that this has occurred despite the lack of research into, and understanding of, the immune elements important for natural host protection against the virus, making this endeavor a somewhat unique one in medical history. In contrast, as pointed out in the review below, there were already important past observations that suggested that respiratory infections at mucosal surfaces were susceptible to immune clearance by mechanisms not typical of infections caused by systemic (blood-borne) pathogens. Accordingly, it was likely to be important to understand the role for both innate and acquired immunity in response to viral infection, as well as the optimum acquired immune resistance mechanisms for viral clearance (B cell or antibody-mediated, versus T cell mediated). This information was needed both to guide vaccine development and to monitor its success. We have known that many pathogens enter into a quasi-symbiotic relationship with the host, with each undergoing sequential change in response to alterations the other makes to its presence. The subsequent evolution of viral variants which has caused such widespread concern over the last 3–6 months as host immunity develops was an entirely predictable response. What is still not known is whether there will be other unexpected side-effects of the deployment of novel vaccines in humans which have yet to be characterized, and, if so, how and if these can be avoided. We conclude by remarking that to ignore a substantial body of well-attested immunological research in favour of expediency is a poor way to proceed.

## 1. Introduction

For the past 18 months, the world has been ravaged by a pandemic caused by a coronavirus infection originating in late 2019 in Wuhan, China. By mid-2020 it became apparent that there had arisen global consensus that the way forward from the socioeconomic and medical morass which had occurred was through rapid development and implementation of a universal vaccination program. However, unlike past precedents, this was to take place in the relative absence of detailed knowledge and investigation into the nature of natural host resistance to the pathogen concerned, and by “speed-tracking” novel vaccine designs to clinical use, again in the absence of detailed knowledge of possible short-term and longer-term implications of this vaccine’s administration. In a recent review the (then) current status of understanding of immunity to SARS-CoV-2, and how this might affect future approaches to protective vaccination, was discussed [1]. The concern was raised in that review that far too little effort had been focused on understanding the nature of the immune response which might provide optimal immune protection. What follows is an analysis of those advances that have taken place to improve that understanding, and how and if this has affected, and may further affect, the global response to the SARS-CoV-2 pandemic.

Mammalian immunity has both an innate and adaptive arm. Protection mediated by innate immunity, the sole immune mechanism for 95% of the species on earth, develops quickly (1–2 days), with some evidence also implying an immune memory, “training”, affording an enhanced protection from reinfection (with the same pathogen) and even enhanced immunity to novel pathogens [2]. This novel idea suggests a much closer parallel with adaptive immunity that has been long thought to be the only immune system to show memory.

**Figure 1:**
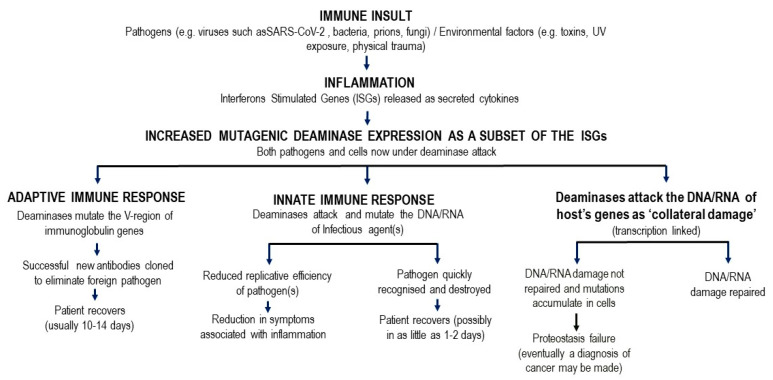
Model linking downstream innate and adaptive immune changes following pathogen insult.

Figure 1 shows the causal links between deaminase mutagenic activity, SARs-Cov-2 infection, the role of interferon stimulated gene (ISG) pathways, the host innate and adaptive immune response, and the subsequent possible accumulation of collateral cell damage. Innate immunity involving the deaminases is designed to inhibit pathogens through multiple, mostly non-genetic, pathways. Genetic targeting of the SARs-Cov-2 RNA genome by deaminases, namely innate immunity-induced mutagenesis of the pathogen genome cripples its replicative efficacy. The deaminases are the main ISG induced proteins that attack the DNA or RNA of invading viruses by extensively mutating their genomes with C-to-U (T) and A-to-I(G) mutations [3,4]. The deaminases APOBEC3B and APOBEC3G, in particular, have been studied for two decades, and they are now colloquially known as ‘virus smashers’ due to their well characterised mechanism of actions that impact viral potency and function [4]. This is the first line of innate immune defence that acts to suppress or eliminate the SARS-CoV-2 virus. During ISG induced attacks on foreign pathogens by deaminases, some *de novo* mutations that remain uncorrected may also accumulate in the DNA of transcribed non-Ig genes, and possibly lead to further cell damage in infected tissue [5].

The mechanism(s) involved in training innate immunity likely involve epigenetic changes (altered DNA methylation; histone deacetylase activity) which results in more rapid activation of the genes implicated in response to pathogens [6]. Epigenetic chemical alterations on sections of the gene make up a part, or all, of the genetic regions that can potentially be targets for deamination during transcription. Conversely, it makes sense that those regions that are chemically protected from deamination are conserved where DNA fidelity needs to be maintained for survival and the proper functioning of an organism. In a landmark study by Guo et al. [7], it was found that the TET1 gene and the oncogenic adenosine deaminase APOBEC1 are actively involved in region-specific neuronal activity-induced DNA methylation changes [7,8]. This concept of training of innate immune responses may in part help explain why infant mortality, and even adult mortality, is less in Bacillus Calmette-Guerin (BCG) vaccinated cohorts (BCG admixed with adjuvants is an excellent inducer of innate immune responses) than in non-vaccinated cohorts from the same population [9]. BCG-mediated training of innate immunity in vaccine development is the founding principle behind the ACTIVATE trial in elderly volunteers to assess the contribution of BCG vaccine in decreasing susceptibility to bacterial disease [10,11] and, more recently, SARS-CoV-2 infection [12].

Defects in innate immunity, as well as acquired immunity, are particularly evident in the elderly [13,14,15,16,17,18]. Innate immunity acts rapidly to control viral replication in infected healthy subjects [19], through type I and type III interferon inducible anti-viral immunity [20]. Elderly patients lacking this rapid innate response are at very high risk for severe outcomes following SARS-CoV-2 infection, including increased morbidity and mortality [21]. Type1 and III interferon inducible genes include APOBEC and ADAR induced expression, which as described in Figure 1 and elsewhere can, in turn, play a role in “haplotype switching” of SARS-CoV-2-expressed genes, leading in turn to the diversification of the virus genetic pattern seen in some subjects, but notably not in those with impaired innate immunity (see below and [22]).

Adaptive (acquired) T and B lymphocyte mediated immunity, while certainly primarily responsible for immunologic memory, takes some 10–14 days post pathogen exposure to become active, but in general shows much greater diversity for pathogen recognition than does innate immunity. Given the experience in understanding how acquired immune mechanisms can be brought to play to enhance pathogen resistance by deliberate vaccination, and the numerous successes evident in global disease control reported as a result, it is not surprising that over the last 12–18 months effort has been directed to making this the key strategy employed against the current pandemic. The discussion below reviews what we have learned about the importance of antibody (B cell mediated) and T effector immunity in providing protection following natural infection, or following vaccination, and how the pathogen has, in turn, responded to naturally acquired or vaccine-induced heightened host resistance. In addition, a brief synopsis of some unexpected adverse effects already noted with vaccines currently “in play”, and how this might affect the future direction of vaccinology will be mentioned.

### 1.1. Heterogeneity in SARS-CoV-2 Antibody Responses and SARS-CoV-2 Protection

Entry of virus into the cells of infected individuals was shown early on to depend on the receptor-binding domain (RBD) of the spike (S) protein of SARS-CoV-2 [23]. Accordingly, much of the research on naturally infected and even vaccinated individuals has focused attention on the epitopes (different unique antigenic configurations) of the RBD recognized by antibodies [24] (and see below, T cells [25]). Even though viral neutralizing titers were low following natural infection and convalescence, a commonality was seen amongst recovered individuals for Ig responses to various RBD domains in the S protein [23]. It is important to note that an independent analysis of naturally infected COVID subjects reported only a very weak correlation between antibody titers and neutralizing activity in sera using commercial clinical laboratory assays [26]. This is perhaps not surprising given an independent study looking at the various B cell subsets giving rise to antigen specific Ig responses following SARS-CoV-2 infection [27]. This group reported that B cells could be segregated into discrete functional subsets specific for the spike (S), nucleocapsid protein (NP), and open reading frame (ORF) proteins (nomenclature accorded, 7a and 8), but only S-specific B cells were enriched in memory B cell clusters, with monoclonal antibodies (mAbs) from these cells being potently neutralizing. In contrast, B cells specific to ORF8 and NP were enriched in naïve and innate-like clusters, and mAbs against these targets were non-neutralizing. Again, studying serum Ig binding across platforms of viral antigens and antibodies with 15 positive and 30 negative SARS-CoV-2 controls followed by viral neutralization assessment S-IgG3 was reported to provide the highest accuracy for predicting serologically positive individuals with virus neutralization activity [28].

The sophistication of dissecting Ig response to the RBD to assess efficacy in protection is highlighted by more recent analysis of epitope binding. Thirty-eight RBD-binding neutralizing Abs with known structures, mostly isolated from virus-infected patients, were grouped into five general clusters, which were, in turn, able to document distinct non-neutralizing faces on the RBD. A maximum of up to 4 of these neutralizing Abs could bind to the RBD simultaneously, with significant implications for vaccine design [29,30]. These clinical analyses highlighting the importance of responses to the RBD in S protein in protection are in turn supported by independent data from animal model studies. Passive transfer of potent neutralizing antibodies (nAbs) to two epitopes on the receptor binding domain RBD of S protein provided protection against disease, as monitored by the maintenance of body weight and low lung viral titers following high-dose SARS-CoV-2 challenge in Syrian hamsters [31]. In addition, mice immunized with a recombinant vaccinia virus expressing a modified SARS-CoV-2-S protein (which was recognized on virally infected cells by anti-RBD Ig and soluble human ACE2 receptor) produced neutralizing Ig which passively protected humanACE2 transgenic mice from lethal SARS-CoV-2 infection [32]. Transgenic mice immunized with vaccinia vector before SARS-CoV-2 infection had no morbidity and weight loss upon intranasal infection with SARS-CoV-2 either 3 wk or 7 weeks later. In addition, there was no detectable infectious SARS-CoV-2 or subgenomic viral mRNAs in the lungs. Further, a greatly reduced induction of cytokine and chemokine mRNAs was reported, with scant levels of virus found in the nasal turbinates of 1/8 rMVA-vaccinated mice on day 2 (and none later) [32].

Despite these data, it should be acknowledged that clinical manifestations of SARS-CoV-2 infection are not the same in children, and nor is the immune response engendered following infection. Children are largely spared from severe respiratory disease but may develop a multisystem inflammatory syndrome similar to Kawasaki’s disease [33]. SARS-CoV-2-specific Igs were less diverse and specific in children compared with adults, with both children and adults producing IgG, IgM and IgA Abs specific for S protein but only adults making significant responses to nucleocapsid (N) protein [33]. Children produced markedly lower neutralizing activity compared to SARS-CoV-2 infected adult cohorts [33]. There is no data yet available on the relative responses of the two cohorts to vaccination.

### 1.2. The Role of Mucosal Immunity in Protection against SARS-CoV-2

It has been known for many years, that the best form of protective immunity for pathogens invading by the nasal or oral route are local secretory IgA responses [34]. Recent analyses on SARS-CoV-2 reinfections and transmissions in vaccinated individuals [35,36] and studies assessing immunization against influenza and SARS-CoV-2 are consistent with this concept [37,38]. Froberg et al. reported that mucosal IgA responses were detected in naturally infected cases in the absence of serum antibody responses, and in this scenario, mucosal antibody levels correlated strongly with virus neutralization. Given the current focus on SARS-CoV-2 vaccination as the primary path forward to resolve the clinical sequelae of the current pandemic, it is concerning that there has been so little attention paid to vaccine-induced mucosal immunity. This may in part at least help explain observations reported in the 20 April 2021 update report from the US Centers for Disease Control on vaccine efficacy [39] which suggested little vaccine protection from infection, though a clear moderation of disease severity in infected vaccinated individuals. An important dichotomy between systemic and mucosal immunity following SARS-CoV-2 infection has been reported by Smithy et al. [40] with important ramifications for treatment, and interpretation of pathology. Independent recent studies by Lopez et al. [41], and Cheemarla et al. [42] show that that the supply of antiviral interferon enables epithelial cells of the nasopharyngeal mucosa to inhibit SARS-CoV-2 growth, with interferon-induced mucosal genes thus serving as biomarkers of infection (see above and below in sections on innate immunity).

A separate study measured humoral responses to SARS-CoV-2, including analysis of the presence of specific neutralizing antibodies in the serum, saliva, and bronchoalveolar fluid of 159 patients following natural infection with SARS-CoV-2. Again, early viral specific humoral responses were dominated by IgA antibodies with peaks during the third week post-infection, with IgA contributing to virus neutralization to a greater extent than IgG or IgM antibodies. While anti-viral IgA serum concentrations decreased after 1 month neutralizing IgA remained detectable in saliva for up to 10 weeks [43]. The same conclusion was reached independently by Butler et al. [44] who acknowledged that while serum neutralization and effector functions correlated with systemic SARS-CoV-2-specific IgG response magnitude, mucosal neutralization was associated with nasal SARS-CoV-2- IgA, along with less severe disease. A recent study has examined the nature of mucosal immunity induced by two independent mRNA vaccines in the USA, BNT162b2 from Pfizer/BioNTech and mRNA-1273 from Moderna [45]. Both vaccines induce antibodies to SARS-CoV-2 S-protein, including neutralizing antibodies (nAbs) to the RBD, with marked increased titers observed following a second dose of vaccine. Again, antibodies to the S-protein and the RBD were reported in saliva samples from mRNA-vaccinated healthcare workers, with 100% of subjects given either vaccine showing IgG in the saliva, and >50% with IgA.

Limited research studies have been reported on vaccine induced mucosal immunity in animals. A chimpanzee adenovirus-vectored vaccine encoding a prefusion stabilized spike protein (ChAd-SARS-CoV-2-S) was studied following intramuscular (im) injection for protection against SARS-CoV-2 infection in mice expressing the human angiotensin-converting enzyme 2 receptor [46]. A single dose induced systemic humoral and cell-mediated immune responses and protected mice against lung infection, inflammation, and pathology, without inducing sterilizing immunity, as confirmed by viral RNA detection after SARS-CoV-2 challenge. In contrast, a single intranasal dose of the same vaccine induced high levels of neutralizing antibodies, enhanced both systemic and mucosal IgA and T cell responses, and prevented SARS-CoV-2 infection in both the upper and lower respiratory tracts [46]. In a study in macaques, a comparison was made between animals receiving both intramuscularly priming and boosting with vaccine and those receiving intramuscularly priming but intranasal boosting with a vaccine. The vaccine used was an adjuvant vaccine with SARS-CoV-2 S protein. While the im only vaccine induced both binding and neutralizing antibody with persistent cellular immunity systemically and mucosal, using a strategy of intranasal boosting resulted in weaker T cell and IgG responses but higher dimeric IgA and IFNα. Following SARS-CoV-2 challenge both groups of animals had no detectable subgenomic RNA in either the upper or lower respiratory tracts compared with naive controls, again supporting the validity of a mucosal immunization strategy [47].

Further insight into the importance of mucosal immunity (and its induction) in effective immunity to SARS-CoV-2 infection comes from studies with children. Unlike other respiratory viruses where disease manifestations are often more severe in children, infection of children with SARS-CoV-2 generally follows a more benign course. Pierce et al. [48] found that SARS-CoV-2 copy numbers, ACE 2, and TMPRSS2 gene expression were similar in children and adults, but infected children had increased expression of molecules indicative of innate immune pathway stimulation (expression of genes associated with IFN signaling, NLRP3 inflammasome, and other innate pathways). Higher levels of IFN-α2, IFN-γ, IP-10, IL-8, and IL-1β proteins were detected in the nasal fluid of children compared with adults, with similar levels of SARS-CoV-2 specific IgA and IgG in nasal fluid of both groups. All children had a far more benign course following infection than the adult cohort. Given the importance of secretory dimeric IgA (sIgA) in protecting mucosal surfaces from pathogens, and evidence (see above) implying the importance of mucosal sIgA in immunity to SARS-CoV-2, of further interest is a report by Quinti et al. on the increased susceptibility and more fulminant course of disease in subjects lacking (genetically) in SARS-CoV-2-specific IgA and secretory IgA [49]. As they note, unlike other primary antibody deficiency entities, selective IgA deficiency is often a “silent” unrecognized condition but may be an (unexplored) important cause of variability in response to SARS-CoV-2 infection. Confirmation of increased susceptibility to SARS-CoV-2 in IgA deficiency has also been reported by Colkesen and colleagues [50].

### 1.3. T Cell Immunity to SARS-CoV-2

It has been known for some time that activated T lymphocytes are crucial for protective immunity to viral infections. This is consistent with what we understand about the quite different antigen recognition by B versus T cells. The latter recognize cell surface MHC-presented epitopes altered following viral infection and thus can act to destroy potential “viral factories” before living viral replication is completed within the infected cell. While monitoring of (serum) Ig may make for easier assessment of the development of an immune response to a pathogen, it may, as inferred above, provide little information about the development of protective immunity in the infected host. The issue is further discussed in some detail in a recent review [51]. Antibody response correlates poorly with disease particularly in mild infections, with a more robust response generally reflective of more severe clinical disease. In contrast, virus-reactive T-cell immunity lasts longer, and natural SARS-CoV-2 infection induces broad epitope coverage, by both CD4 and CD8 T cells. There is less restriction to S protein immunity than for Ig responses, though any correlation with disease outcome remains to be determined. Correlation of clinical outcomes with laboratory markers of cell-mediated immunity, not only with antibody response, may shed further light on how to optimize induction of protective immunity after both natural infection and vaccination. A preliminary report of just such an investigation was recently published [52] for subjects aged 18–55 years, up to 8 weeks after vaccination with a single dose of ChAdOx1 nCoV-19. CD4 T cell responses were characterized by interferon-γ and tumor necrosis factor-α cytokine secretion, with predominantly IgG1 and IgG3 antibodies. Some CD8^+^ T cells were also induced of monofunctional, polyfunctional and cytotoxic phenotypes, with, to date, little documented clinical significance. A more exhaustive study of CD8 T cell immunity following natural infection was also reported recently [53]. A highly heterogeneous response was seen across numerous CD8+ epitopes and across multiple (6) HLAs, with up to 52 unique epitopes recorded, against both structural and non-structural targets in the SARS-CoV-2 proteome, though again any correlation with the outcome has yet to be made [53].

Some attempt to gain more insight into the mechanism(s) of T cell resistance comes from animal studies by Zhuang and co-workers [54]. Their data indicate that the type I interferon pathway was critical for generating optimal antiviral T cell responses after SARS-CoV-2 infection of mice, and that T cell vaccination alone could even provide partial protection from severe disease in infected animals.

## 2. How Does the Clinical Efficacy of SARS-CoV-2 Vaccines Align with Induction of Immunity?

### 2.1. Innate Immunity

In the introduction [2,3,4,5,6,7,8,9,10,11,12] mention was made of the potential, largely unexplored, role of innate immunity in SARS-CoV-2 infection. Innate immunity is triggered by a family of so-called pattern recognition receptors, and is known to induce interferons and multiple cytokines, activating cells of both the myeloid and lymphoid differentiation pathway for protection against pathogens [2]. Live-attenuated vaccines for tuberculosis, measles, and polio have all been shown to “train” the innate immune system, through histone modifications and epigenetic reprogramming of monocytes to develop an inflammatory phenotype, thus enhancing broad resistance to other infectious diseases, of which SARS-CoV-2 infection may be an example [55,56].

A recent study comparing innate immune response to Influenza and SARS-CoV-2 in nasal washes from infected adults suggested an important difference in innate immunity following SARS-CoV-2 infection [57], with decreased IFN-associated transcripts in neutrophils, macrophages and epithelial cells compared with influenza-infected individuals, and decreased epithelial cell-cell interactions. GWAS studies have also implied an important link between the IFN pathway and disease severity [58]. In an important new publication, Inanova et al. [59] compared various immune parameters in subjects post natural infection or SARS-CoV-2 vaccination (SARS-CoV-2 BNT162b2 mRNA). Both infection and vaccination induced innate and adaptive immune responses, but only in SARS-CoV-2 infected patients, and not vaccinated individuals, was this characterized by augmented interferon responses. This was in turn correlated with the upregulation of cytotoxic genes in the peripheral T cells and innate-like lymphocytes in the same cohort. In addition, as assessed by B and T cell receptor repertoires, in SARS-CoV-2 infected patients most clonal B and T cells in infected patients were effector cells, while in vaccinated subjects the expanded cells were primarily circulating memory cells. Further complicating attempts to understand immune protection following deliberate vaccination are data that we have summarized elsewhere suggesting natural herd immunity was already developing on population scales across Europe before the vaccine rollout had really begun [60]. A recent publication suggests another promising approach to elevate broad spectrum intra-nasal anti-viral Innate Immunity in the upper respiratory tract, using local delivery of an engineered defective viral genome which ultimately leads to enhanced both local and distal type I interferon responses [61].

### 2.2. B Cell Immunity following Vaccination

Studies in a naturally infected cohort of individuals who had recovered from mild SARS-CoV-2 infection showed evidence for SARS-CoV-2 specific IgG, neutralizing antibodies, and memory B and memory T cells persisting beyond months [62]. Memory T cells secreted cytokines and expanded upon antigen re-encounter, and memory B cells expressed receptors capable of neutralizing virus when expressed as monoclonal antibodies. Similarly, Dan et al. reported memory cell survival beyond 8 months in both B and T cells following infection [63], though it seemed B cell memory responses were more persistent than T cell immunity, although the clinical significance of this was not addressed. This observation on long-term persistence of neutralizing IgG following natural infection is consistent with studies from a German cohort assessed up to 9 months post-infection [64] and other studies showing persistent viral neutralizing antibody correlated with outcome [65], and even that viral rebound after early clearance is associated with lower induction and lower levels of RBD-specific IgA and IgG antibodies [66].

How does the development of a protective Ig response compare after natural infection versus vaccination? In particular, what implications are there for vaccine induced immunity to infection, given the (relatively) rapid antigenic drift in SARS-CoV-2 reported over the last 8 months [67]. Mutations on the S protein, in particular, can, in theory, affect binding to either (or both) of the cell receptor ACEII or antibody binding. A shared mutation that increases binding to ACEII, and transmissibility is present in the variants B.1.1.7, (UK) P.1, (Brazil); and B.1.351, (South Africa). The B.1.351 and P.1 variants also display another mutation which decreases binding of neutralizing antibodies, leading to (partial) immune escape and favoring reinfections [67]. The contribution of a background of increased immunity (in the “at risk” population) to the emergence of new mutations remains to be explored. A recent analysis of publicly available genomic sequence and epidemiological data during the 2nd Wave in Victoria, Australia discusses the likelihood of rapid amplification of SARS-CoV-2 in the face of a failed innate immune response (as in the elderly co-morbid patients). Thus these publicly available data already suggest that capricious expansion of a common genomic sequence is favored with limited further putative deaminase-mediated mutation at APOBEC and ADAR-deaminase motifs (C-sites, A-sites) in the SARS-CoV-2 genomes isolated from such patients [68].

Analysis of antibody and memory B cell responses of a cohort of 20 volunteers given either of two mRNA vaccines against SARS-CoV-2 showed the plasma neutralizing activity and relative numbers of RBD-specific memory B cells was similar in vaccinated and naturally infected cohorts. However, activity against SARS-CoV-2 variants was reduced significantly [69]. A similar reduction, albeit small, in neutralizing activity against the B.1.1.7 SARS-CoV-2 and in binding to the RBD motif was reported by Collier et al. [70] after vaccination with a mRNA-based vaccine, with more substantial loss of neutralizing activity following introduction of a second variant in the B.1.1.7 background, which they hypothesize may represent a threat to the efficacy of this vaccine. Similar concerns are raised by reports from other groups [71,72]. ACEII binding and neutralizing Ab, isolated following natural infection with a number of different SARS-CoV-2 variants, also highlighted preservation of RBD binding to some more conserved sites.

A more comprehensive summary using 506,768 SARS-CoV-2 genome isolates, including mutations of the S-RBD from patients, was reported to explore the efficacy of immunity to the growing list of SARS-CoV-2 variants, also concluded that most variants were associated with increased binding to ACEII, and thus likely greater infectiousness. Many new RBD mutants were characterized which could affect neutralizing Ig binding to the RBD, including mutations now described in the California variant B.1.427, and the Mexico variant B.1.1.222, which markedly enhances the infectivity of the latter. The authors conclude that “the genetic evolution of SARS-CoV-2 on the RBD, which may be regulated by host gene editing, viral proofreading, random genetic drift, and natural selection, (can) give rise to more infectious variants that will potentially compromise existing vaccines and antibody therapies” [73,74]. A similar concern was raised by Venkatakrishnan et al. [75]. Despite this gloomy forecast, however, there is clear evidence that the current vaccines in use have led to a clinically significant protective response to infection in those at risk [76,77], as was emphasized in a recent report exploring hospitalization post-vaccination [78].

### 2.3. T Cell Immunity following Vaccination

As mentioned previously, it seems self-evident that a systematic analysis of T cell epitopes recognized by subjects following natural infection, correlated with disease outcome, is essential to guide interpretation of monitoring patient responses, and to develop vaccines that might prove efficacious in protection. A USA government-led clinical trial [79] was designed with just this in mind in 2020 (now in data analysis phase, but yet to report). Blood samples from SARS-CoV-2 infected patients who have recovered from the infection were screened using a genome-wide, high-throughput screening technology [80], with the hope that identification of T cell receptors and immunogenic viral epitopes on SARS-CoV-2 which may contribute to development of long-lasting protection against SARS- CoV-2 will be achieved. Preliminary reports from other groups, using a more restricted research design, already hold out hope that these studies will have some utility. Thus a longitudinal analysis (up to 6 months post-infection) revealed decreasing S and nucleocapsid-specific antibody responses while in contrast functional T cell responses remained persisted and even increased, over the same period, with many dominant T cell epitopes identified [81]. A more recent genome-wide screen approach was used to explore CD8 immunity in convalescent SARS-CoV-2 individuals [82]. From a pool of ~3140 MHC Class I-binding peptides covering the complete SARS-CoV-2 genome over 120 immunogenic peptides were identified, with a subset of these found to represent immunodominant SARS-CoV-2 T cell epitopes. A pre-existing T cell recognition signature was seen in naïve individuals, possibly reflecting exposure to previous coronavirus infections. More importantly, a robust T cell activation profile could be characterized in previously infected patients, which was most marked in those with severe disease, and there was a minimal response seen in those with mild disease or in naïve (uninfected) individuals [82].

Further studies have attempted to explore the nature of recognition of viral variants in vaccinated/infected versus naïve subjects, in order to compare the data with that observed from comparison of Ig responses in similar groups (see above). Subjects receiving either Pfizer-BioNTech (BNT162b2) or Moderna (mRNA-1273) mRNA-based SARS-CoV-2 vaccine were characterized, and found to show broad T cell responses to the SARS-CoV-2-S protein, with only 4/23 targeted peptides potentially affected by mutations in the UK (B.1.1.7) and South African (B.1.351) variants. CD4+ T cells from vaccine recipients recognized the 2 variant spike proteins as effectively as they recognized the ancestral virus S-protein from the ancestral virus, in contrast to antibody data discussed above. Interestingly, a 3-fold increase in the CD4+ T cell responses to influenza S-peptides was seen after vaccination, implying a cross-protection (following SARS-CoV-2 vaccination) against some endemic coronaviruses [83]. Others have also reported that SARS-CoV-2-specific T cells from vaccinated individuals recognize variant SARS- CoV-2 isolates and that that vaccinated convalescents have more persistent nasopharynx-homing SARS- CoV-2- specific T cells compared to infection-naïve counterparts [84]. However, mention should be made of a conflicting report by Gallagher et al. [85] which used standard functional assays to assess T-cell immunity to SARS- CoV-2 in uninfected, convalescent, and vaccinated individuals. While vaccinated individuals showed stronger T-cell responses to the wild-type spike and nucleocapsid proteins, compared with convalescent patients, quite diminished T-cell responses to spike variants (B.1.1.7, B.1.351, and B.1.1.248) were observed in vaccinated but otherwise healthy donors, in parallel to the Ig data discussed before. Other than differences in the assays used, acknowledging again an absence of any correlation with clinical utility, there is no obvious explanation for the discrepancies with the studies reported in [85] vs. [81,82,83,84].

The importance of understanding T cell immunity to SARS-CoV-2 is confirmed by a recent report investigating the logistics of using in vitro expanded SARS-CoV-2 immune T cells in adoptive immunotherapy in immunocompromised subjects [86]. This group expanded SARS- CoV-2 specific T cells from convalescent donors using GMP facilities and combinations of membrane, spike, and nucleocapsid peptides. All induced IFN-γ production, in 27 (59%), 12 (26%), and 10 (22%) respectively in convalescent donors, and in 2 of 15 unexposed controls. Polyfunctional CD4-restricted T-cell epitopes were identified within a conserved region of membrane protein, which induced polyfunctional T-cell responses. The authors suggest these may have utility in the development of both effective vaccine and T-cell therapies for use in immunocompromised patients with blood disorders or following bone marrow transplantation. An exhaustive review of current candidate vaccines in phase 3 trial has recently been published [87].

## 3. Unexpected Adverse Effects of SARS-CoV-2 Vaccination

It seems apropos to conclude the present discussion with some thoughts on adverse effects noted from SARS-CoV-2 vaccination. Heralding the rapid and novel introduction of mRNA vaccines into the clinical armamentarium, a new formulation of synthetic mRNA strands encoding the SARS-CoV-2-S glycoprotein, packaged in lipid nanoparticles to deliver mRNA to cells, Verbeke et al. suggest we stand on the threshold of a “new dawn” in vaccinology [88]. However, as they acknowledge, there are still huge gaps in our understanding. Already data from the two widely used mRNA vaccines BNT162b2 and mRNA-1273, suggest that the nucleoside-modified mRNA approach allows for delivery of higher maximal tolerable doses and might thus in part explain why these, rather than the adenovirus encoded S-protein in a more conventional vaccine, allows for more rapid generation of antibody responses [88]. It remains unexplained why the two similar (nucleoside-modified) mRNA vaccines elicited quite different S-specific CD8^+^ T cell responses. There are a number of plausible hypotheses, including, but not limited to, possible differences in innate responses to the two candidates; and the mRNA sequence design (e.g., UTR inclusion, codon optimizations) which might have contributed to the potency and reactogenicity (minor adverse effects) of the vaccines. It is beyond doubt, that more in-depth knowledge on the in vivo delivery efficiency and the particular innate immune effects of the different mRNA vaccines will contribute to the design of even safer and more effective mRNA vaccines in the future.

The need to address these issues is highlighted by the recent CDC briefing, held with the plan to alleviate many concerns within the public over what may be (in the longer-scheme of things) minor issues [89]. As a brief summary they emphasize:

- Although still not fully understood, even the most effective of vaccines does not prevent illness 100% of the time-vaccine breakthrough occurs, though generally with less disease severity.
- Since it takes some 10–14 days post-vaccination to develop immunity, if an individual was infected shortly before/after vaccination, it is predicted they may still become infected. In addition (see above) infection may occur with a variant for which the current vaccines are not providing effective immunization for.
- While to date no unusual patterns have been detected in the data of people infected post-vaccination, CDC has an ongoing goal to identify any unusual patterns, such as trends in age or sex, the vaccines involved, underlying health conditions, and whether particular SARS-CoV-2 variants are causing sickness.
- There is abundant data now to suggest that vaccines help protect people who are vaccinated from getting COVID-19 or from getting severely ill from SARS-CoV-2. Nevertheless, because people can still get sick and possibly spread the virus to others after being fully vaccinated, the current recommendation remains that people continue to take simple public health measures to protect themselves and others-see also [90]. Indeed, the efficacy of vaccination for protection against documented infection re-mains a controversial issue [91].

Others have focused attention on clearly documented adverse effects from SARS-CoV-2 vaccines already in use. First and foremost, amongst these is vaccine-induced immune thrombotic thrombocytopenia (VITT) in the aftermath of vaccination with the adenoviral vector SARS-CoV-2 vaccine ChAdOx1 nCoV-19 [92]. Rare patients (less than 1 in 100,000) develop thrombosis and thrombocytopenia 5–24 days after vaccination, often with thromboses at unusual sites (cerebral venous sinus; portal, hepatic and splanchnic veins), test strongly positive in PF4/polyanion enzyme immunoassays (EIAs), and show serum-induced platelet activation which is maximal in the presence of PF4. It is not clear what components of the vaccine are responsible for the enhanced response to an unrelated host protein (PF4), and why it occurs only after exposure to the adenovirus vector. It may be that PF4 is a bystander component within an immune complex that activates platelets. Thiele et al. assessed the frequency of anti-PF4/polyanion antibodies in healthy vaccinees and assessed whether PF4/polyanion EIA-positive sera exhibited platelet-activating properties after vaccination with ~140 each of ChAdOx1 nCoV-19 or BNT162b2 (BioNTech/Pfizer) vaccines [93]. Although 19 of 281 participants tested positive for anti-PF4/polyanion antibodies post-vaccination (All: 6.8% [95%CI, 4.4–10.3]; BNT162b2: 5.6% [95%CI, 2.9–10.7]; ChAdOx1 nCoV-19: 8.0% [95%CI, 4.5–13.7%]), none of the PF4/polyanion EIA-positive samples induced platelet activation in the presence of PF4. They concluded that positive PF4/polyanion EIAs could occur after SARS-CoV-2 vaccination with both mRNA- and adenoviral vector-based vaccines, but the majority of these antibodies likely had minor (if any) clinical relevance. The pathogenic platelet-activating antibodies causing VITT did not occur commonly after vaccination.

Additional groups have focused on the theoretical risk associated with the current vaccines, arguing that their “rush into service” has ignored potential concerns with their use, particularly the concern regarding induction of autoimmune reactivity [94]. As an example, the failure of SARS vaccines in animal trials involved pathogenesis consistent with an immunological priming that could involve autoimmunity in lung tissues due to previous exposure to the SARS spike protein [95]. A comparison of immunogenic epitopes in SARS-CoV-2-S proteins, and other SARS-CoV-2 proteins with human protein to search for homologous matching. The author concluded that only one immunogenic epitope in SARS-CoV-2 had no homology to human proteins, and that many of the overlaps with human proteins could theoretically help explain some of the symptoms associated with the pathogenesis of SARS-CoV-2. In a similar vein, Lu et al. asked whether the rapid move to market the current SARS-CoV-2 vaccines might leave us at risk of causing neurologic disorders like those previously recognized, including vaccine-related demyelinating diseases, fever-induced seizure, and other deficits [96]. Others have focused on the issue of myocarditis following SARS-CoV-2 infection and/or vaccination [97], and other more subtle autoimmune type responses following SARS-CoV-2 infection [98]. It is self-evident, by comparison with past experience, that only time will tell how significant a problem this represents.

## 4. Concluding Remarks

In conclusion, we recall that pandemics of the type we have experienced over the past 18 months are far from new. Similar pandemics have punctuated human history over many millennia, often contributing to episodes of severe attenuation of populations, and in some instances leading even to the collapse of empires. In the past, however, the societal role for coping with such pandemics has been limited-confined in the main to alleviating suffering that resulted from the disease, and perhaps enabling the segregation of infective victims wherever possible. There was obviously no possibility at this stage of any globally coordinated response, nor of any large-scale intervention, to minimize transmission and thus accelerate the end of a pandemic. The advantage we possess now is scientific knowledge of the type we have discussed in this paper. If such knowledge is utilized honestly and dispassionately, a new utopia beckons; otherwise, we may be no better off now than in earlier epochs, and by some reckoning perhaps even worse.

All pandemics are of course self-limiting, eventually ending through the development of herd immunity to the infective agent. The global response to the current pandemic, involving severe and often punitive limitations of individual freedom is clearly unprecedented in history. The justification for such draconian measures is based on the assertion that scientific advances have made available a shortcut to the natural process of immunity that would greatly reduce the total number of infections, and the number of deaths. We show in this review that some aspects of the scientific arguments being currently deployed are either deficient or seriously flawed.

## 5. Summary

There is hope that we are now approaching an entrenchment phase in our response to the SARS-CoV-2 pandemic, with more widespread uptake of vaccines, protection of vulnerable population cohorts (especially the elderly), and adherence to better public health measures continuing to improve the overall outlook. At all levels, politically, sociologically, ethically, scientifically and medically, there have been instances of major mismanagement and misunderstanding, coupled with gross errors of judgement, which have clearly cost lives. As reviewed above, it can be argued that we still have failed to recognize the importance of implementation of basic science knowledge, both new research and understanding old observations, which even now would likely improve the future course of the disease. We need to remain vigilant in the face of having implemented so many previously untried and untested therapies for the appearance of new signs and symptoms in treated patients which are early indications of adverse events, for which VITT may be merely the tip of a large iceberg. As the philosopher, George Santayana once said “Those who cannot remember the past are condemned to repeat it”. We need to make sure that the valuable lessons learned, at all levels, over the last 18 months are not forgotten.
